# Study on the Effect of the Envelope of Terahertz Unipolar Stimulation on Cell Membrane Communication-Related Variables

**DOI:** 10.34133/research.0755

**Published:** 2025-07-15

**Authors:** Wenfei Bo, Rong Che, Feng Jia, Kai Sun, Qiang Liu, Lemeng Guo, Xiaobo Zhang, Yubin Gong

**Affiliations:** ^1^ College of Information and Communication, National University of Defense Technology, Wuhan 430000, China.; ^2^ School of Electronic Science and Engineering, University of Electronic Science and Technology of China, Chengdu 611731, China.

## Abstract

The development of terahertz science and technology has shown new application prospects in artificial intelligence. Terahertz stimulation can lead to information communication of cells. Terahertz unipolar picosecond pulse train stimulation can activate cell membrane hydrophilic pores and protein ion channels. However, the effect of the envelope of the terahertz unipolar stimulation remains unknown. This paper studies the effect of the envelope on membrane communication-related variables and the accompanying energy consumption by a cell model with considerations of hydrophilic pores and Na^+^, K^+^-ATPase. According to the results, terahertz unipolar picosecond pulse train stimulation can deliver the signal contained in its envelope into the variation rates of membrane potentials no matter whether the hydrophilic pores are activated or not and also into the variation rates of the ion flow via the pores after activation of the pores. In contrast, the ion flow via Na^+^, K^+^-ATPase seems irrelevant to the signal in the envelope. Moreover, the ion flows show a modulation effect on the variation rates of membrane potentials. The accompanying power dissipations in the cases of different envelopes are similar, as low as around the level of 10^−11^ W. The results lay the foundations for application in artificial intelligence, like brain–machine communications.

## Introduction

With the ongoing development and applications of artificial intelligence (AI) in various fields, current AI suffers many challenges [1–7]. The capabilities and qualities of AI in power consumption, information transmission, and processing for intelligent problems like those in which high-level cognition, or perceptual–motor tasks, etc., is involved, are far lower than those of human brain intelligence [1,4]. In AI research, brain–machine communication is one of the frontier development directions [8–10]. The information transfer rates required for the real-time transmission of whole encephalic information with all types of human senses that human brain intelligence deals with simultaneously exceed 100 Gb/s [11], which is far beyond the state-of-the-art information transfer rates of both invasive and noninvasive brain–machine communication techniques [11–14].

Currently, the development of terahertz science and technology has revealed that terahertz waves can be generated, be transmitted, and activate critical cell membrane ion channels in nerve cells [15–21]. Notably, it is found that the myelin sheath possesses an around 2-fold higher refraction index compared to the outer medium or the inner axon [15], and it makes the myelin sheath a dielectric waveguide suitable for signal propagation in the mid-infrared to terahertz spectral range, and the energy of signal propagation can be supplied and amplified at the nodes of Ranvier [15]. Those implicate the approaching to the reveal of the mechanism of high-efficiency intelligent information transmission and processing and low power consumption of human brain intelligence and show new application prospects of terahertz science and technology in AI like brain–machine communications [15–19,22].

**Fig. 1.**
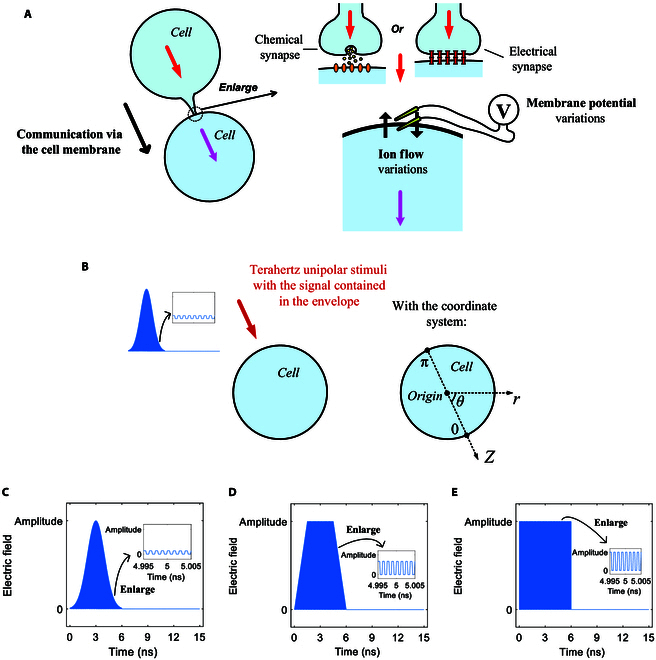
(A) Communication via the cell membrane, during which membrane potential and transmembrane life ion flow are critical variables related to the cell membrane. By the variations of membrane potential and ion flow after neurotransmitter release of chemical synapses [23,42] or the direct action of electrical synapses [41,43] or the action of both [41,43], the signal (marked by a red arrow) can be transmitted to the cell (marked by a pink arrow). (B) Stimulation of a terahertz unipolar picosecond pulse train with the signal in the envelope (marked by a dark red arrow) to the cell. The Gauss envelope is shown here for an example. The cell is placed in a spherical coordinate system, where *z* is parallel to the terahertz electric field, *r* is the radial direction that is the normal vector of the cell membrane at any position, and *θ* is the polar angle that is the angle between the *z* axis and the *r* axis in the range of 0 to *π*. (C to E) Schematic illustrations of 6-ns, 0.8-THz unipolar picosecond pulse trains with different envelopes. The inset in each illustration is the enlarged view of the pulse train in the time domain at an arbitrary instant, for example, around 5 ns. (C) Gauss envelope. (D) Trapezoid envelope. (E) Rectangular envelope.

The ion transports via nerve cell membrane ion transport pathways as well as the accompanying generated electromagnetic signals are the bases for the information transmission and processing of human brain intelligence [23–26] and the information communication via brain–machine interfaces [11–13,27,28]. Terahertz stimulation can lead to information communication of cells due to the cause of transmembrane life ion flows [17,18,20,21,29–35]. It is found that terahertz unipolar picosecond pulse train stimulation can activate cell membrane hydrophilic pores [36,37] and protein ion channels like Na^+^, K^+^-ATPase [22]. The activation of hydrophilic pores and protein ion channels leads to the variations of transmembrane life ion flows and membrane potentials with low energy consumption of ATPase [22,38–40]. The behaviors of Na^+^, K^+^-ATPase and energy consumption in the ion flow of hydrophilic pores under terahertz unipolar stimulation are numerically investigated, and it is shown that Na^+^, K^+^-ATPase can be activated and stay activated for a while before closing with a power dissipation of around 10^−11^ W in 2 types of cells, i.e., neurons and myocytes, in a narrow frequency band of 0.1 to 1.2 THz [22]. Moreover, the membrane potential variations in a cell under terahertz unipolar stimulation with a special signal, i.e., a triangle envelope, are investigated, and the investigation results show that during the variations of the membrane potentials, the variation rates of the membrane potentials contain the triangle envelope signal [40]. Cell membrane potentials and transmembrane life ion flows are the basic variables of biological cells that are closely correlated to cell membrane information communication [18,23,41–43], for example, as shown in Fig. 1A. However, it remains unknown the effect of the signal contained in the terahertz unipolar picosecond pulse train on those cell membrane communication-related variables and accompanying power dissipations.

In this paper, the effects of the signal contained in the envelope of the stimulation are numerically investigated. The theoretical modeling of the relationships of the cell membrane communication-related variables and accompanying power dissipation to the envelope of terahertz unipolar stimulation and the numerical methods are described in Methods. The results are presented in Results and are discussed and concluded in Discussion and Conclusion, respectively.

## Results

The cell is under terahertz unipolar stimulation with signals in its envelope, and then the stimulation is switched off, as illustrated in Fig. 1B. The stimulation is of Gaussian, trapezoid, or rectangular envelope lasting for 6 ns, whose electric field waveforms are illustrated in Fig. 1C to E, respectively. After the stimulation, the numerical calculations continue for 9 ns. The standard error for the results is zero in the statistical tests because the results are the same in each simulation repeat.

### Effect of the envelope on membrane potentials

#### Variations of membrane potentials by stimulation with a Gaussian envelope

**Fig. 2.**
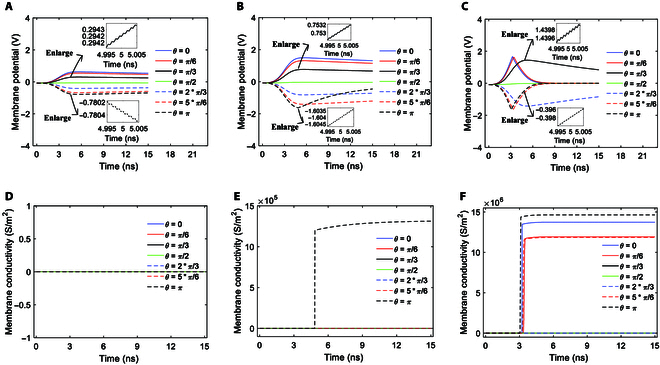
Membrane potentials at *θ* = 0, *π*/6, *π*/3, *π*/2, 2*π*/3, 5*π*/6, and *π* versus time in the case of 6-ns, 0.8-THz unipolar stimulation with Gauss envelopes at amplitudes of 0.9 × 10^7^ (A), 2 × 10^7^ (B), and 3.68 × 10^7^ V/m (C). Membrane conductivities at *θ* = 0, *π*/6, *π*/3, *π*/2, 2*π*/3, 5*π*/6, and *π* versus time in the case of 6-ns, 0.8-THz unipolar stimulation with Gauss envelopes at amplitudes of 0.9 × 10^7^ (D), 2 × 10^7^ (E), and 3.68 × 10^7^ V/m (F).

As we can see from Fig. 2, under stimulation, the membrane potentials increase first and then decrease with different extents at each polar angle *θ* except *θ* = *π*/2. Obviously, those variations can be divided into 2 groups. The first is a steep increase followed by a much slower decrease toward zero, including all *θ* except *θ* = *π*/2 in Fig. 2A; *θ* = 0, *π*/6, *π*/3, 2*π*/3, and 5*π*/6 in Fig. 2B; and *θ* = *π*/3 and 2*π*/3 in Fig. 2C. The second is a steep increase followed by a steep decrease, including *θ* = *π* in Fig. 2B and *θ* = 0, *π*/6, 5*π*/6, and *π* in Fig. 2C. From Fig. 2D to F, it can be seen that the membrane permeability conductivities caused by the hydrophilic pores are different at different angles *θ*, and thus, the pores are not uniformly distributed on the sphere of the membrane. By comparison of Fig. 2A to C with Fig. 2D to F, it is apparent that the difference in the variations between those 2 groups is attributed to the activation of cell membrane hydrophilic pores in the second group. In the first group, the hydrophilic pores are hardly ever activated, as shown in Fig. 2D to F. In contrast, in the second group, the activation of hydrophilic pores induces massive transmembrane ion flows [22,39], which accelerate the decrease in membrane potentials toward zero, thus leading to the difference.

**Fig. 3.**
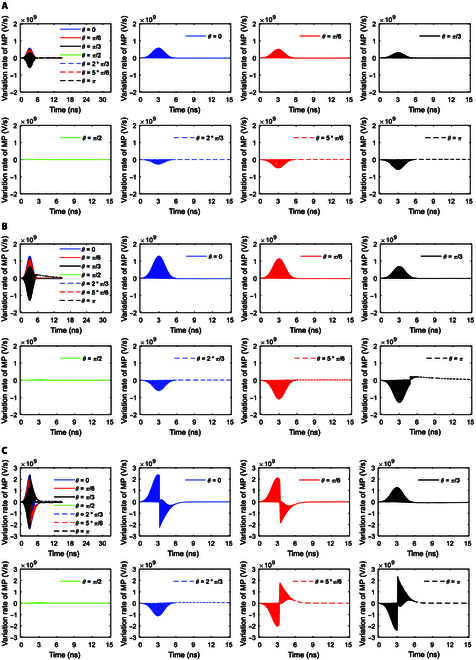
(A) Variation rates of membrane potentials (MPs) at *θ* = 0, *π*/6, *π*/3, *π*/2, 2*π*/3, 5*π*/6, and *π* versus time in the case of 6-ns, 0.8-THz unipolar stimulation with Gauss envelopes at an amplitude of 0.9 × 10^7^ V/m. (B) Variation rates of MPs at *θ* = 0, *π*/6, *π*/3, *π*/2, 2*π*/3, 5*π*/6, and *π* versus time in the case of 6-ns, 0.8-THz unipolar stimulation with Gauss envelopes at an amplitude of 2 × 10^7^ V/m. (C) Variation rates of MPs at *θ* = 0, *π*/6, *π*/3, *π*/2, 2*π*/3, 5*π*/6, and *π* versus time in the case of 6-ns, 0.8-THz unipolar stimulation with Gauss envelopes at an amplitude of 3.68 × 10^7^ V/m.

Moreover, as shown in the insets in Fig. 2A to C, the membrane potentials vibrate at a terahertz frequency in the processes of those variations during the stimulation (from 0 to 6 ns), and additionally, the vibration is more marked in the first group than in the second group. The reasons are as follows: The terahertz unipolar picosecond pulse train is composed of many picosecond pulses (*g*_(*T*/2)_(*t*) = 1 in Eq. (1)) separated by picosecond intervals (*g*_(*T*/2)_(*t*) = 0 in Eq. (1)), as seen in the insets in Fig. 1C to E. The picosecond pulses lead to marked changes in membrane potentials (*g*_(*T*/2)_(*t*) = 1 in Eq. (7)), and contrarily, the picosecond intervals themselves lead to seldom change in the potentials (*g*_(*T*/2)_(*t*) = 0 in Eq. (7)). The difference in the induced changes in membrane potentials between the pulses and the intervals leads to the vibration of the membrane potentials at a terahertz frequency. Additionally, because the activation of hydrophilic pores in the second group leads to marked change in membrane potentials, which masks the vibration, the vibration is less marked in the first group. The induced changes in membrane potentials are further depicted by the variation rates of membrane potentials, as shown in Fig. 3.

Figure 3A shows the variation rates of membrane potentials with respect to time at an amplitude of 0.9 × 10^7^ V/m. Due to the relatively low amplitude and short duration of the stimulation in this case, the terahertz unipolar stimulation does not activate cell membrane hydrophilic pores (see Fig. 2D). As we can see from Fig. 3A, the variation rates have Gaussian envelopes during the stimulation. Furthermore, the amplitudes of the Gaussian envelopes are larger near the 2 poles of the cell at *θ* = 0 and *π* than near the equator of the cell at *θ* = *π*/2.

In the case of 2 × 10^7^ V/m terahertz unipolar stimulation, hydrophilic pores are activated in the cell membrane at *θ* = *π* and are not activated at *θ* = 0, *π*/6, *π*/3, *π*/2, 2*π*/3, and 5*π*/6 (see Fig. 2E). Similar to the case of 0.9 × 10^7^ V/m terahertz unipolar stimulation, the variation rates of membrane potentials have Gaussian envelopes at *θ* = 0, *π*/6, *π*/3, *π*/2, 2*π*/3, and 5*π*/6 (see Fig. 3A and B). The difference takes place at *θ* = *π* where the hydrophilic pores are activated. As we can see, before the activation of the hydrophilic pores at *θ* = *π* (see around <4.8 ns in Fig. 2E), the envelope of the variation rates in the case of 2 × 10^7^ V/m is similar to that in the case of 0.9 × 10^7^ V/m (see Fig. 3A and B). After the activation of the hydrophilic pores (around >4.8 ns), the envelope in the case of 2 × 10^7^ V/m shows obvious difference from that in the case of 0.9 × 10^7^ V/m at *θ* = *π* (see Fig. 3A and B). Although the envelope maintains the part of the Gaussian envelope, the baseline that the envelope mounts on is not at 0 any longer but has a positive value that has a tendency to decrease toward 0 (see Fig. 3B). This is the modulation effect of massive transmembrane ion flows *I_p_* induced by the activation of hydrophilic pores. Because *I_p_* is added to the variation rates of the membrane potential as a baseline according to Eq. (8), the activation of hydrophilic pores changes the baseline from zero to a nonzero value, and the baseline varies with the variation of the ion flows *I_p_*, that is, a tendency to decrease toward 0 (see the Na^+^ ion flow via the pores in the case of 2 × 10^7^ V/m in the section after next section, for example). Moreover, because *I_p_* is added in the form of its opposite number from Eq. (8), the sign of the baseline is opposite to the sign of the variation rates that are prior to the activation of hydrophilic pores, and in this case, it is positive.

Furthermore, the hydrophilic pores are activated in a larger range of the cell membrane and are activated earlier in the case of 3.68 × 10^7^ V/m than in the case of 2 × 10^7^ V/m (see Fig. 2F and E). Therefore, the hydrophilic pores are activated at more polar angles (*θ* = 0, *π*/6, 5*π*/6, and *π* in Fig. 3C) in comparison with that at *θ* = *π* in the case of 2 × 10^7^ V/m (Fig. 3B), and the time duration of the Gaussian envelope at those polar angles before the activation becomes shorter, but the time duration of the Gaussian envelope that mounts on the nonzero baseline becomes longer (see Fig. 3B and C). Likewise, the envelopes of the variation rates at *θ* = *π*/3, *π*/2, and 2*π*/3 where the hydrophilic pore is not activated are Gaussian, which are similar to those at all *θ* in the case of 0.9 × 10^7^ V/m and at *θ* = 0, *π*/6, *π*/3, *π*/2, 2*π*/3, and 5*π*/6 in the case of 2 × 10^7^ V/m (see Fig. 3).

#### Variations of membrane potentials by stimulation with trapezoid and rectangular envelopes

**Fig. 4.**
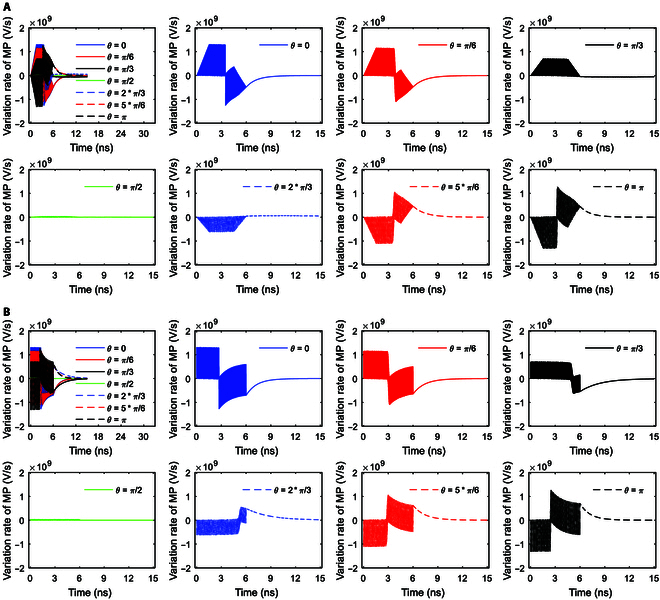
(A) Variation rates of MPs at *θ* = 0, *π*/6, *π*/3, *π*/2, 2*π*/3, 5*π*/6, and *π* versus time in the case of 6-ns, 0.8-THz unipolar stimulation with trapezoid envelopes at an amplitude of 2 × 10^7^ V/m. (B) Variation rates of MPs at *θ* = 0, *π*/6, *π*/3, *π*/2, 2*π*/3, 5*π*/6, and *π* versus time in the case of 6-ns, 0.8-THz unipolar stimulation with rectangular envelopes at an amplitude of 2 × 10^7^ V/m.

Figure 4A and B respectively show the variation rates of membrane potentials under 0.8-THz unipolar stimulation with trapezoid and rectangular envelopes in the case of 2 × 10^7^ V/m. By comparing Fig. 4A and B with Fig. 3B, it can be seen clearly that the relationships of the variation rates of membrane potentials with the envelope of the stimulation are the same as those in the case of a Gaussian envelope both before and after the activation of hydrophilic pores. That is to say, under terahertz unipolar stimulation with a trapezoid envelope, the envelope of the variation rates of membrane potentials is a trapezoid or part of the trapezoid mounting on the zero baseline at a polar angle *θ* when hydrophilic pores are not activated. Furthermore, the envelope of the variation rates of membrane potentials becomes part of the trapezoid mounting on the nonzero baseline that has a tendency to decrease toward zero at *θ* when hydrophilic pores are activated. Likewise, under stimulation with a rectangular envelope, the envelope of the variation rates of membrane potentials is rectangular or part of the rectangle mounting on the zero baseline at *θ* when hydrophilic pores are not activated. Moreover, the envelope of the variation rates of membrane potentials is part of the rectangle mounting on the nonzero baseline that tends toward zero at *θ* when hydrophilic pores are activated.

Furthermore, it is clear from Figs. 3B and 4A and B that the envelope of the variation rates of membrane potentials reflects the envelope of the terahertz unipolar picosecond pulse train stimulation. That means that the variations of membrane potentials can reflect the signals carried in the envelope of terahertz unipolar stimulation.

### Effect of the envelope on transmembrane life ion flows

#### Effect on ion flows via hydrophilic pores

**Fig. 5.**
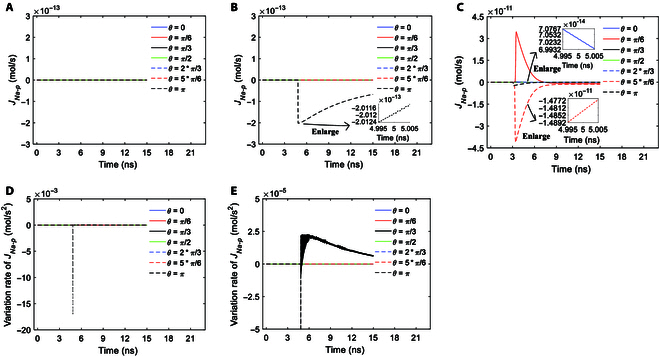
The transmembrane Na^+^ flow via hydrophilic pores at *θ* = 0, *π*/6, *π*/3, *π*/2, 2*π*/3, 5*π*/6, and *π* versus time in the case of 6-ns, 0.8-THz unipolar stimulation with Gauss envelopes at amplitudes of 0.9 × 10^7^ (A), 2 × 10^7^ (B), and 3.68 × 10^7^ V/m (C). The variation rates of the transmembrane Na^+^ flow via hydrophilic pores at *θ* = 0, *π*/6, *π*/3, *π*/2, 2*π*/3, 5*π*/6, and *π* versus time in the case of 6-ns, 0.8-THz unipolar stimulation with Gauss envelopes at an amplitude of 2 × 10^7^ V/m (D). (E) is an enlarged view of (D) in the vertical axis.

**Fig. 6.**
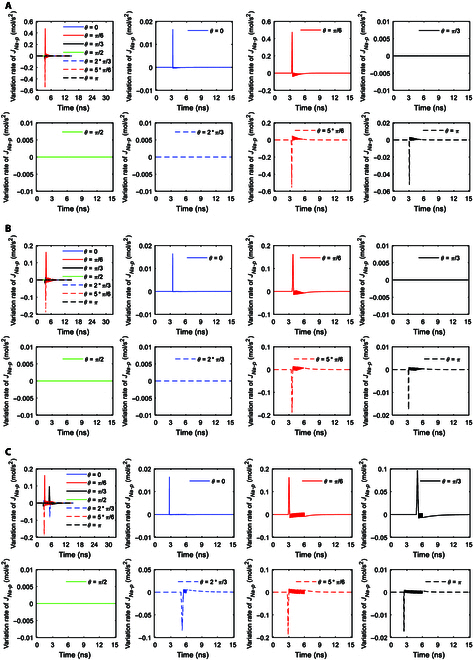
(A) Variation rates of the transmembrane Na^+^ flow via hydrophilic pores at *θ* = 0, *π*/6, *π*/3, *π*/2, 2*π*/3, 5*π*/6, and *π* versus time in the case of 6-ns, 0.8-THz unipolar stimulation with Gauss envelopes at an amplitude of 3.68 × 10^7^ V/m. (B) Variation rates of the transmembrane Na^+^ flow via hydrophilic pores at *θ* = 0, *π*/6, *π*/3, *π*/2, 2*π*/3, 5*π*/6, and *π* versus time in the case of 6-ns, 0.8-THz unipolar stimulation with trapezoid envelopes at an amplitude of 2 × 10^7^ V/m. (C) Variation rates of the transmembrane Na^+^ flow via hydrophilic pores at *θ* = 0, *π*/6, *π*/3, *π*/2, 2*π*/3, 5*π*/6, and *π* versus time in the case of 6-ns, 0.8-THz unipolar stimulation with rectangular envelopes at an amplitude of 2 × 10^7^ V/m.

In comparison of Figs. 5A to C and 2D to F, we can see that before the activation of hydrophilic pores, the ion flow via the pores is zero. Moreover, at the time when the hydrophilic pores are activated, there is a sudden increase in the ion flow from 0. This is due to the sudden increase in membrane conductivity at the time that hydrophilic pores are activated (see Fig. 2D to F). After the activation, the increase rate of the membrane conductivity slows down and the conductivity becomes nearly steady soon (see Fig. 2D to F). When the conductivity is nearly steady, the variations of the ion flows via hydrophilic pores are nearly exclusively a result of the variations of membrane potentials, and then the ion flows decrease as the membrane potentials decrease. That is why the variation tendencies of the ion flows shown in Fig. 5A to C are similar to the variation tendencies of membrane potentials shown in Fig. 2A to C after the activation. Meanwhile, the variations of the ion flows are different during the pulses and the intervals of the terahertz unipolar picosecond pulse train, which leads to the slight vibration of the ion flows at a period of picosecond, which corresponds to a terahertz frequency, during the stimulation, as shown in the insets in Fig. 5A to C. The induced changes in ion flows are further depicted by the variation rates of the ion flows, as shown in Figs. 5D and E and 6A.

As we can see in Figs. 5D and E and 6A, the variation rates of ion flows remain zero at *θ* when hydrophilic pores are not activated. At the time that hydrophilic pores are activated, the variation rates have a large peak. This is because of the sudden increase in the ion flows from 0 to maxima. After that, similar to the case of membrane potentials, the variation rates of ion flows have part of the Gaussian envelope mounting on a nonzero baseline, which has a tendency to decrease toward 0 during the stimulation. This is because the ion flows nearly exclusively vary with membrane potentials after the activation of hydrophilic pores.

As we can see from Fig. 6B and C, the variation rates of ion flows have part of the trapezoid envelope and rectangular envelope, respectively, under the stimulations with trapezoid and rectangular envelopes.

#### Effect on ion flows via Na^+^, K^+^-ATPase

**Fig. 7.**
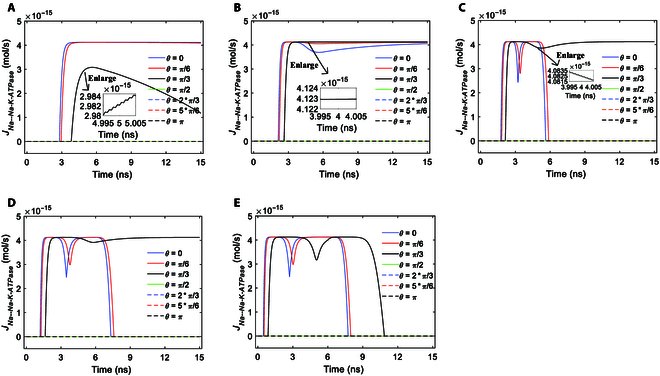
The transmembrane Na^+^ flow via Na^+^, K^+^-ATPase at *θ* = 0, *π*/6, *π*/3, *π*/2, 2*π*/3, 5*π*/6, and *π* versus time in the case of 6-ns, 0.8-THz unipolar stimulation with different envelopes at different amplitudes. (A) Gauss envelope at an amplitude of 0.9 × 10^7^ V/m. (B) Gauss envelope at an amplitude of 2 × 10^7^ V/m. (C) Gauss envelope at an amplitude of 3.68 × 10^7^ V/m. (D) Trapezoid envelope at an amplitude of 2 × 10^7^ V/m. (E) Rectangular envelope at an amplitude of 2 × 10^7^ V/m.

Since the ion concentration variations are nearly ignorable for Na^+^ and K^+^ ions (see Fig. S1), the activation and the ion flow variations of Na^+^, K^+^-ATPase are mainly caused by the variations of membrane potentials, which are induced by both the electric field of terahertz unipolar stimulation in Eq. (1) and the ion flows via hydrophilic pores in Eq. (11). The corresponding Na^+^ ion flows of Na^+^, K^+^-ATPase as the membrane potentials vary under the stimulation with Gaussian envelopes of 3 different amplitudes in Fig. 2A to C are shown in Fig. 7A to C.

**Fig. 8.**
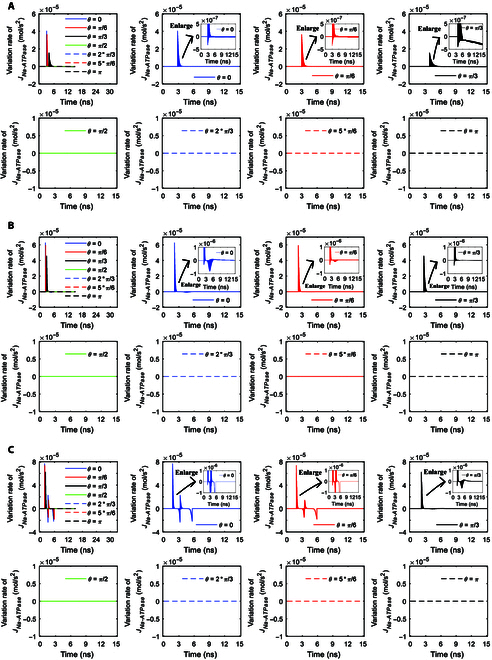
(A) Variation rates of the transmembrane Na^+^ flow via Na^+^, K^+^-ATPase at *θ* = 0, *π*/6, *π*/3, *π*/2, 2*π*/3, 5*π*/6, and *π* versus time in the case of 6-ns, 0.8-THz unipolar stimulation with a Gauss envelope at an amplitude of 0.9 × 10^7^ V/m. The insets are the enlarged views in vertical axes. (B) Variation rates of the transmembrane Na^+^ flow via Na^+^, K^+^-ATPase at *θ* = 0, *π*/6, *π*/3, *π*/2, 2*π*/3, 5*π*/6, and *π* versus time in the case of 6-ns, 0.8-THz unipolar stimulation with a Gauss envelope at an amplitude of 2 × 10^7^ V/m. The insets are the enlarged views in vertical axes. (C) Variation rates of the transmembrane Na^+^ flow via Na^+^, K^+^-ATPase at *θ* = 0, *π*/6, *π*/3, *π*/2, 2*π*/3, 5*π*/6, and *π* versus time in the case of 6-ns, 0.8-THz unipolar stimulation with a Gauss envelope at an amplitude of 3.68 × 10^7^ V/m. The insets are the enlarged views in vertical axes.

It can be seen from Fig. 7A that under stimulation at an amplitude of 0.9 × 10^7^ V/m, the ion flows at *θ* = 0 and *π*/6 increase steeply to the maximum and then they stay nearly invariant, and the ion flows at *θ* = *π*/3 increase to the maximum and then decrease. At a larger amplitude of 2 × 10^7^ V/m seen from Fig. 7B, the ion flows at *θ* = *π*/3 increase to the maximum and then stay nearly invariant instead of markedly decreasing, and in addition, ion flows at *θ* = 0 show an extraordinary opposite-direction-varying peak at around 6 ns due to the inhibition effect [22,32]. At a further larger amplitude of 3.68 × 10^7^ V/m seen from Fig. 7C, the ion flows at *θ* = 0 and *π*/6 show a larger extraordinary opposite-direction-varying peak at around 3 ns due to the inhibition effect, and the ion flows at *θ* = *π*/3 also show an extraordinary opposite-direction-varying peak at around 6 ns. Thus, the larger the amplitude, the larger the extraordinary opposite-direction-varying peak due to the inhibition effect. Likewise, the induced changes in ion flows are further depicted by the variation rates of the ion flows, as shown in Fig. 8.

The variation rates of the Na^+^ ion flows of Na^+^, K^+^-ATPase with respect to time under stimulation at an amplitude of 0.9 × 10^7^ V/m are shown in Fig. 8A. It can be seen from the figure that the envelopes of the variation rates show sudden increases to maxima followed by gradual decreases to zero at *θ* = 0 and *π*/6. As the duration of the increase to the maximum is longer at *θ* = *π*/3 than that at *θ* = 0 and *π*/6 (see Fig. 7A), the duration of the sudden increase followed by a gradual decrease is longer at *θ* = *π*/3 (see Fig. 8A). Moreover, the baseline after that duration has a negative value instead of being zero at *θ* = *π*/3 (see Fig. 8A), because of the obvious decrease in the Na^+^ ion flows after reaching the maximum (see Fig. 7A).

In the case of 2 × 10^7^ V/m terahertz unipolar stimulation as shown in Fig. 8B, the envelopes of the variation rates of the Na^+^ ion flows show a sudden increase followed by a gradual decrease at *θ* = 0, *π*/6, and *π*/3 at first, which is similar to that at *θ* = 0, *π*/6, and *π*/3 in the case of 0.9 × 10^7^ V/m. However, after that (around time >3 ns in Fig. 7B), the Na^+^ ion flows at *θ* = 0 and *π*/6 do not become invariant but have slight extraordinary opposite-direction-varying peaks due to the slight inhibition effect, so the variation rates have corresponding envelopes for these at these angles (see Fig. 8B). In contrast, since the Na^+^ ion flow at *θ* = *π*/3 becomes nearly invariant after around time >3 ns (see Fig. 7B), the variation rate is nearly 0 then at *θ* = *π*/3 (see Fig. 8B), which is similar to that at *θ* = 0 and *π*/6 in the case of 0.9 × 10^7^ V/m (see Fig. 8A).

Furthermore, in the case of 3.68 × 10^7^ V/m terahertz unipolar stimulation as shown in Fig. 8C, at *θ* = *π*/3, there is a slight inhibition effect similar to that at *θ* = 0 and *π*/6 in the case of 2 × 10^7^ V/m terahertz unipolar stimulation, so the variation rates have a similar envelope for this after a sudden increase followed by a gradual decrease (around time >3 ns). At *θ* = 0 and *π*/6, after the increase to the maxima, the ion flows have a marked extraordinary opposite-direction-varying peak at around 3 ns due to the strong inhibition effect (see Fig. 7C). Therefore, the variation rates at *θ* = 0 and *π*/6 have a marked negative envelope followed by a marked positive envelope at around 3 ns (see Fig. 8C). After that, the ion flows at *θ* = 0 and *π*/6 decrease to zero at around 6 ns as the membrane potentials decrease. Hence, at around 6 ns, the variation rates at *θ* = 0 and *π*/6 decrease to negative maxima followed by a sudden return to zero (see Fig. 8C).

**Fig. 9.**
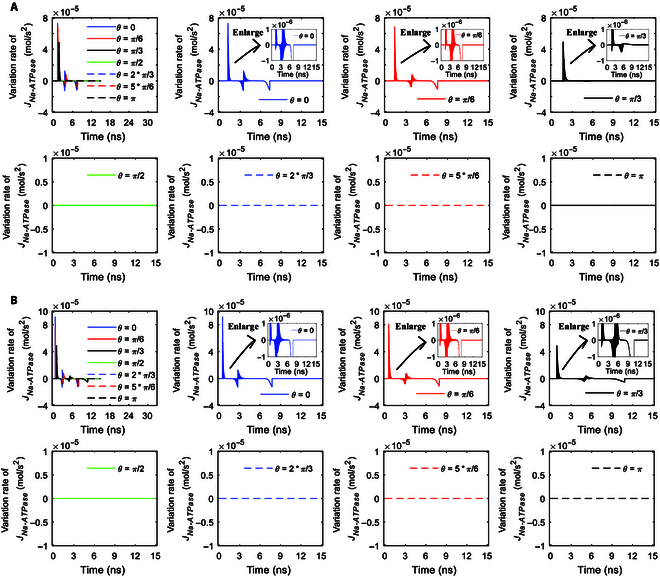
(A) Variation rates of the transmembrane Na^+^ flow via Na^+^, K^+^-ATPase at *θ* = 0, *π*/6, *π*/3, *π*/2, 2*π*/3, 5*π*/6, and *π* versus time in the case of 6-ns, 0.8-THz unipolar stimulation with a trapezoid envelope at an amplitude of 2 × 10^7^ V/m. The insets are the enlarged views in vertical axes. (B) Variation rates of the transmembrane Na^+^ flow via Na^+^, K^+^-ATPase at *θ* = 0, *π*/6, *π*/3, *π*/2, 2*π*/3, 5*π*/6, and *π* versus time in the case of 6-ns, 0.8-THz unipolar stimulation with a rectangular envelope at an amplitude of 2 × 10^7^ V/m. The insets are the enlarged views in vertical axes.

Figure 7D and E show the Na^+^ ion flows of Na^+^, K^+^-ATPase under terahertz unipolar stimulation with trapezoid and rectangular envelopes. It is clear that most of the nonzero ion flows have marked extraordinary opposite-direction-varying peaks. Then, the variation rates at those *θ* as shown in Fig. 9A and B have the corresponding 3 parts, which are similar to these at *θ* = 0 and *π*/6 in Fig. 8C in the case of a Gaussian envelope. One exception of the nonzero ion flows is at *θ* = *π*/3 in Fig. 7D, which has a slight extraordinary opposite-direction-varying peak. Then, the variation rates at *θ* = *π*/3 in Fig. 9A have the corresponding 2 parts, which are similar to those at *θ* = *π*/3 in Fig. 8C in the case of a Gaussian envelope.

It is clear that the Na^+^, K^+^-ATPase ion flows as well as their variation rates seem to not reflect the envelopes of the terahertz unipolar stimulation.

### Effect of the envelope on power dissipation

**Fig. 10.**
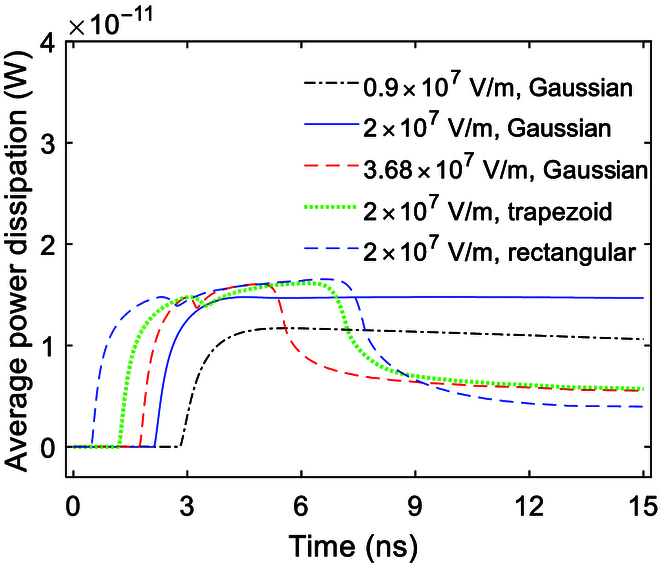
Average power dissipation of Na^+^, K^+^-ATPase in the case of Gauss envelopes at amplitudes of 0.9 × 10^7^, 2 × 10^7^, and 3.68 × 10^7^ V/m and in the case of trapezoid and rectangular envelopes at an amplitude of 2 × 10^7^ V/m.

From Fig. 10, it can be seen that the accompanying power dissipations are similar in the cases of Gaussian, trapezoid, and rectangular envelopes, as low as around the level of 10^−11^ W. Therefore, it might mean that the power dissipations remain nearly stable when stimulating with different signals by terahertz unipolar picosecond pulse trains.

## Discussion and Conclusion

**Fig. 11.**
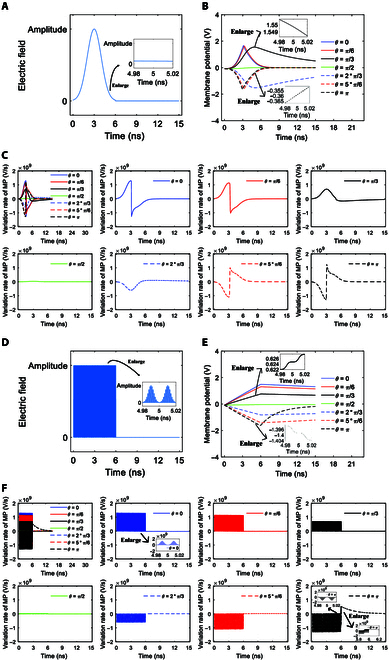
(A) Schematic illustration of a 6-ns Gauss pulse, which is the envelope in Fig. 1C. (B) MPs at *θ* = 0, *π*/6, *π*/3, *π*/2, 2*π*/3, 5*π*/6, and *π* versus time in the case of 6-ns Gauss pulse stimulation at an amplitude of 2 × 10^7^ V/m. (C) Variation rates of MPs at *θ* = 0, *π*/6, *π*/3, *π*/2, 2*π*/3, 5*π*/6, and *π* versus time in the case of 6-ns Gauss pulse stimulation at an amplitude of 2 × 10^7^ V/m. (D) Schematic illustration of a 6-ns, 0.8-THz unipolar picosecond pulse train whose envelope is a 0.02-ns-duration Gauss pulse train. (E) MPs at *θ* = 0, *π*/6, *π*/3, *π*/2, 2*π*/3, 5*π*/6, and *π* versus time in the case of 6-ns, 0.8-THz unipolar picosecond pulse train stimulation whose envelope is a 0.02-ns-duration Gauss pulse train at an amplitude of 2 × 10^7^ V/m. (F) Variation rates of MPs at *θ* = 0, *π*/6, *π*/3, *π*/2, 2*π*/3, 5*π*/6, and *π* versus time in the case of 6-ns, 0.8-THz unipolar picosecond pulse train stimulation whose envelope is a 0.02-ns-duration Gauss pulse train at an amplitude of 2 × 10^7^ V/m. The insets are the enlarged views in the time domain.

This study focuses on the effects of the envelope of terahertz unipolar stimulation on cell membrane communication-related variables, membrane potentials, and transmembrane life ion flows, with considerations of the hydrophilic pores and Na^+^, K^+^-ATPase. It is worth mentioning that the interaction of the electromagnetic stimulation at terahertz frequencies and the natural vibration motions in cell membrane molecules might have influence on the ion flows, and then the conductance in Eq. (8) and the diffusion coefficients in Eqs. (11) and (12) might be influenced. For example, molecular dynamics simulations show that the conductance is 4 times at 0.1 THz for a KcsA channel due to the effects of terahertz waves [44]. However, the conductance in Eq. (8) and the diffusion coefficients in Eqs. (11) and (12) play trivial roles in the effect of the envelope of terahertz unipolar stimulation on cell membrane communication-related variables, and the possible changes in those parameters due to the effects of terahertz waves have insignificant influences (see the Supplementary Materials). In the literature [22], it is shown that the effects in a narrow terahertz range from 0.1 to 1.2 THz are almost the same in the case of the same amplitudes of the electric field in vacuum of a terahertz pulse train, and it remains unknown whether the effects at frequencies other than this terahertz range 0.1 to 1.2 THz are the same or not. Moreover, since the dielectric constant varies with respect to frequency, the same electric field in vacuum at different frequencies within 0.1 to 1.2 THz corresponds to different macroscopic electric fields. Therefore, although the terahertz unipolar stimulation contains the dc component, it does not indicate that the primary effect of the terahertz unipolar stimulation within this narrow terahertz range is due to the dc electric field and is unrelated to terahertz. Besides the influence on the dielectric constant that relates the macroscopic electric field to the electric field in vacuum, the terahertz pulse train’s role is further studied by stimulation of only the envelope signal, i.e., a 6-ns Gauss pulse (see Fig. 11A), and making comparison with the case under stimulation in Fig. 1C. By comparisons of Fig. 11B and C and Figs. 2A to C and 3A to C, it is clear that the membrane potentials do not vibrate and the Gaussian shape is reflected directly by the shape of the variation rate of the membrane potentials instead of by the shape of the envelope of the variation rate of the membrane potentials because of the lack of the vibration of the membrane potentials when the stimulation of only the envelope signal is applied. Therefore, under the stimulation of the terahertz unipolar picosecond pulse train with signals contained in the envelope, the envelope signals determine the shape of the envelope of the variation rates of the membrane potentials, and the terahertz pulse train induces the vibration of the membrane potential with a frequency the same as the stimulation frequency and it plays the role of a signal carrier. In addition to the 6-ns envelope signals (see Fig. 1C to E), the signals carried on the envelope of the stimulation can be any other waveforms, for example, 0.02-ns-duration Gauss pulses (see Fig. 11D to F), as long as they can be modulated on the amplitude of the terahertz pulse train. The development of detection techniques for cell membrane communication-related variables in picosecond time resolution is necessary for experimental measurements, such as the application of the terahertz time-domain spectroscopy technique. Moreover, the simulation methods in this study can be applied when the wavelength of the terahertz stimulation is far larger than the cell sample and can be used for outputs of membrane potential and ion flow variations with the inputs of the cell size and terahertz unipolar stimulation parameters.

Terahertz unipolar picosecond pulse train stimulation can induce the vibration of cell membrane potentials at a terahertz frequency and deliver the signal contained in its envelope into the variation rates of the membrane potentials for further signal processing and transmission by cells’ physiological processes. This is additionally a potential way of transferring information from terahertz stimulation into biological cells. The effect of terahertz unipolar picosecond pulse train stimulation being able to activate cell membrane Na^+^, K^+^-ATPase and hydrophilic pores [22,36,37] and the characteristic of terahertz unipolar picosecond pulse train stimulation being able to deliver its signals into cell membrane potentials make terahertz unipolar picosecond pulse train stimulation have versatile applications, which are beyond applications as end/reset signals [22] and are achieved by varying its electromagnetic parameters, such as amplitude.

In conclusion, signals can be contained in the envelopes of a terahertz unipolar picosecond pulse train by amplitude modulation. By modulation with Gaussian, trapezoid, and rectangular pulses, a terahertz unipolar picosecond pulse train can have Gaussian, trapezoid, and rectangular envelopes. The effects of the signal contained in the envelope of the stimulation on cell membrane communication-related variables, the membrane potentials and transmembrane life ion flows, and the accompanying power dissipations are theoretically studied.

Under stimulation with a terahertz unipolar picosecond pulse train, cell membrane potentials vary accordingly. During their variations, the membrane potentials vibrate at a terahertz frequency. Furthermore, the variation rates of the membrane potentials reflect the envelope of the terahertz unipolar stimulation. That is to say, under stimulation with a Gaussian envelope, the envelope of the variation rates of the membrane potentials is Gaussian. Moreover, under stimulation with trapezoid and rectangular envelopes, the envelopes of the variation rates are trapezoidal and rectangular, respectively.

After the activation of cell membrane hydrophilic pores, the envelope of the variation rates of the ion flows via hydrophilic pores can reflect part of the envelope of the terahertz unipolar stimulation. Moreover, at this time, the envelopes of the variation rates for both the membrane potentials and the ion flows via hydrophilic pores have nonzero baselines, as a result of the modulation effect of the massive transmembrane ion flows caused by the activation of hydrophilic pores.

Furthermore, after the activation of cell membrane Na^+^, K^+^-ATPase, the envelope of the variation rates of the ion flows via Na^+^, K^+^-ATPase shows a close correlation with the amplitude of the stimulation. Moreover, the relationships to the amplitude of the stimulation seem similar in the cases of the stimulations of Gaussian, trapezoid, and rectangular envelopes.

The power dissipations of Na^+^, K^+^-ATPase show no big difference and remain nearly stable at an extremely low level when stimulating with Gaussian, trapezoid, and rectangular signals by means of terahertz unipolar picosecond pulse trains.

This study contributes to uncovering the mechanism of nerve cell membrane information transmission and processing in which terahertz electromagnetic signals are involved and lays the basis for the application of terahertz science and technology in AI, like brain–machine communications.

## Methods

### Signals contained in the envelope of a terahertz unipolar picosecond pulse train

As for an amplitude-modulated terahertz unipolar picosecond pulse train, the signals can be contained in its envelope. Its electric field is given by

$$E\left(t\right)=E_{\mathrm{env}}\left(t\right)\cdot \sum_{n=-\infty }^{\infty }g_{\left(T/2\right)}\left(t-n\cdot T\right)$$ (1)

where *T* is the period of the unipolar picosecond pulse train, *n* is an integer, and *g*_(*T*/2)_(*t* − *n* · *T*) is a single unipolar picosecond pulse at time *t* = *n* · *T*. *g*_(*T*/2)_(*t*) is a gate function that can be expressed by

$$g_{\left(T/2\right)}\left(t\right)=\left\{\begin{array}{cc} 1, & \left|t\right|<T/4\\ 0, & \left|t\right|>T/4 \end{array}\right.$$ (2)

*E_env_*(*t*) is the envelope. In the case of a Gaussian envelope, it is

$$E_{\mathrm{env}\_\text{Gauss}}\left(t\right)=E_{m}\cdot \text{exp}\left[-{\left(t-\mathrm{PW}/2\right)}^{2}/2/{\left(\mathrm{PW}/6\right)}^{2}\right]$$ (3)

where *E_m_* is the amplitude of the stimulation. *PW* is the stimulation duration; in this study, the electric field *E_env_Gauss_*(*t*) is truncated and neglected at *t* < 0 and *t* > *PW* when the electric field is less than 1.11% of the amplitude *E_m_*.

In the case of a trapezoid envelope, it is

$$\begin{array}{c} E_{\mathrm{env}\_\text{trape}}\left(t\right)=\left\{\begin{array}{cc} 4E_{m}/\mathrm{PW}\cdot t, & 0\leq t<\mathrm{PW}/4\\ E_{m}, & \mathrm{PW}/4\leq t\leq 3\cdot \mathrm{PW}/4\\ \;-4E_{m}/\mathrm{PW}\cdot \left(t-\mathrm{PW}\right), & \;3\cdot \mathrm{PW}/4<t\leq \mathrm{PW} \end{array}\right. \end{array}$$ (4)

In the case of a rectangular envelope, it is

$$E_{\mathrm{env}\_\text{rect}}\left(t\right)=E_{m}\cdot g_{\mathrm{PW}}\left(t-\mathrm{PW}/2\right)$$ (5)

### Cell membrane potentials under terahertz unipolar stimulation with signals contained in the envelope

The terahertz unipolar picosecond pulse train containing signals in its envelope stimulates a cell with a 6.6-μm diameter. Then, the membrane potential of the cell is given by the superposition principle of electric field as

$$V_{m}=V_{m\_\text{physiol}}+V_{m\_\mathrm{THz}}$$ (6)

where *V_m_physiol_* is the cell physiological membrane potential and *V_m_THz_* is the membrane potential induced by the stimulation. *V_m_THz_* has a positive correlation with the electric field of the stimulation, which can be written as

$$V_{m\_\mathrm{THz}}∼C_{1}\cdot E\left(t\right)\cdot \text{cos}\theta$$ (7)

where *C*_1_ is a constant and *θ* is the polar angle in the spherical coordinate system and denotes the angle between the terahertz electric field (*z* axis) and the normal vector of cell membrane (the radial line *r*), where the geometric center of the cell is at the origin in the spherical coordinate system, as shown in Fig. 1B.

Based on the principle of transmembrane current continuity at the whole cell membrane, in which the capacitive current is taken into consideration, the variation rate of membrane potential with time is

$$\frac{\partial V_{m}}{\partial t}=-\frac{g_{1}}{C_{m}}\left(V_{m}-V_{\text{rest}}\right)-\frac{I_{p}}{C_{m}}$$ (8)

where *g*_1_ is the membrane conductance of total physiological ion channels, *C_m_* is the membrane capacitance, *V_rest_* is the physiological resting membrane potential, and *I_p_* is the total current of life ions via hydrophilic pores.

The shortest wavelength of the terahertz electromagnetic wave of the terahertz unipolar stimulation in the cellular aqueous environment is far larger than the size of the cell (diameter of 6.6 μm) and also larger than the cell system in the simulation (diameter of 19.8 μm) in this study, so it is a quasi-magnetostatic problem. Therefore, due to the negligible phase difference of the electromagnetic field of the terahertz unipolar stimulation within the cell system, the electric field of the terahertz unipolar stimulation is approximately uniform spatially. Then, it follows that the electric field of the terahertz unipolar stimulation is an approximately irrotational field. Furthermore, the electric field that forms the physiological resting membrane potential is also an irrotational field. Then, the electric fields in intracellular and extracellular environments satisfy the Laplace equation of scalar electrical potential Φ as

$$\left\{\begin{array}{c} \nabla^{2}\Phi_{i}=0\\ \nabla^{2}\Phi_{o}=0 \end{array}\right.$$ (9)

where Φ*_i_* and Φ*_o_* are respectively intracellular and extracellular electrical potentials. The electric field is the negative gradient of the scalar electrical potential Φ, i.e., −∇Φ. The terahertz pulses have influences on the electrical potentials Φ in the cell system and further have influences on the charge distribution and potential distribution, and the influences are reflected mainly on the membrane potential owing to the electric neutrality in bulk solution in intracellular and extracellular environments [34]. The membrane potential can be calculated by

$$V_{m}=\Phi_{i}-\Phi_{o}$$ (10)

As we can see from the above equations, the membrane potential is a function of both terahertz electric fields and transmembrane life ion flows.

### Transmembrane ion flows and power dissipations under stimulation with signals contained in the envelope

The transmembrane flow of each type of ion via hydrophilic pores is formulated by a generalized modified Poisson–Nernst–Planck model [39,45] as

$$J_{j\_\mathrm{hp}}=-\frac{D_{j}c_{j}{\mathrm{Fz}}_{j}}{R_{u}T}\cdot \frac{-V_{m}}{d_{m}}-D_{j}\nabla c_{j}-\frac{D_{j}c_{j}\sum_{j=1}^{N}N_{A}a_{j}^{3}\nabla c_{j}}{1-\sum_{j=1}^{N}N_{A}a_{j}^{3}c_{j}}$$ (11)

where *D_j_* is the diffusion coefficient of the *j* type of ions, *c_j_* is the ion concentration, *F* is the Faraday constant, *Z_j_* is the valence, *R_u_* is the gas constant, *T* is the Kelvin temperature (309 K), *d_m_* is the membrane thickness, *N_A_* is the Avogadro constant, and *a_j_* is the effective ion radius. The membrane thickness is 5 nm according to the thickness of the phospholipid bilayer [46]. The effective ion radii for Na^+^, K^+^, Ca^2+^, and Cl^−^ are respectively 3.58, 3.31, 4.12, and 3.32 Å from experimental measurements [47–49]. The diffusion coefficients for Na^+^, K^+^, and Cl^−^ are respectively 1.33 × 10^−9^, 1.96 × 10^−9^, and 2.07 × 10^−9^ m^2^/s based on experimental measurements [49–51], and that for Ca^2+^ is 1.4 × 10^−9^ m^2^/s based on experimental measurements [52,53].

Then, the variation rate of the ion flow with time is

$$\begin{array}{c} \frac{\partial J_{j\_\mathrm{hp}}}{\partial t}=\frac{\partial }{\partial t}\left(\frac{D_{j}c_{j}{\mathrm{Fz}}_{j}V_{m}}{R_{u}{\mathrm{Td}}_{m}}\right)\\ \;-\frac{\partial }{\partial t}\left(D_{j}\nabla c_{j}\right)-\frac{\partial }{\partial t}\left(\frac{D_{j}c_{j}\sum_{j=1}^{N}N_{A}a_{j}^{3}\nabla c_{j}}{1-\sum_{j=1}^{N}N_{A}a_{j}^{3}c_{j}}\right) \end{array}$$ (12)

where *c_j_o_* and *c_j_i_* are the extracellular and intracellular ion concentrations of the *j* type of ions, respectively.

From Eqs. (11) and (12), since the intracellular and extracellular ion concentrations are nearly invariant with time especially during the stimulation [22,39], the variation rate of the ion flow via hydrophilic pores is primarily dominated by the membrane potentials, which are correlated to the electric field of the terahertz unipolar pulse train. The hydrophilic pores activated by the terahertz unipolar pulse train containing signals in the envelope are evaluated by the Neu and Krassowska electroporation model [22,37,39,49].

In addition, the transmembrane ion flows via Na^+^, K^+^-ATPase is formulated by the Na^+^, K^+^-ATPase model [22,54]. The Na^+^ ion flow is given by

$$J_{\mathrm{Na}\_\text{ATPase}}=3F_{c}\cdot v_{\mathrm{cyc}}\left(V_{m}\right)/q_{\mathrm{ele}}/N_{A}$$ (13)

where *F_c_* is a factor, *q_ele_* is the elementary charge, *v_cyc_*(*V_m_*) is the steady-state cycle rate of ATPase, which is a function of membrane potential *V_m_*.

The K^+^ ion flow is related to the Na^+^ ion flow according to the stoichiometry of Na^+^, K^+^-ATPase ion transport, and can be expressed by

$$J_{K\_\text{ATPase}}=-2/3\cdot J_{\mathrm{Na}\_\text{ATPase}}$$ (14)

Then, the variation rate of the ion flow is related to the steady-state cycle rate *v_cyc_*,

$$\frac{\partial J_{\mathrm{Na}\_\text{ATPase}}}{\partial t}=3F_{c}/q_{\mathrm{ele}}/N_{A}\cdot \frac{\partial }{\partial t}\left[v_{\mathrm{cyc}}\left(V_{m}\right)\right]$$ (15)

The power dissipation of Na^+^, K^+^-ATPase is a function of the ion flow *J_Na_ATPase_*. After substituting *J_Na_ATPase_* with Eq. (13), the power dissipation is expressed by

$$P_{\text{ATPase}}=Q_{\mathrm{ATP}}\cdot F_{c}\cdot v_{\mathrm{cyc}}\left(V_{m}\right)/q_{\mathrm{ele}}/N_{A}$$ (16)

where *Q_ATP_* is the hydrolysis energy of one adenosine triphosphate molecule, which is equal to 10 · *k_B_T*.

According to Eqs. (13) to (16), the ion flow via Na^+^, K^+^-ATPase and the accompanying power dissipations are also functions of membrane potentials.

### Numerical methods

In order to study the relationships of the cell membrane communication-related variables membrane potentials and transmembrane life ion flows and the accompanying power dissipations with the envelope of the terahertz unipolar stimulation, equations are simultaneously solved in a spherical coordinate system numerically by finite difference in time domain.

Membrane potentials are calculated numerically by Eqs. (6) and (8) to (10). In the calculation of the membrane potentials, the initial condition Φ*_i_* − Φ*_o_* = *V_rest_* is applied. Furthermore, under the stimulation of the terahertz unipolar pulse train with signals in the envelope, the boundary condition Φ*_o_* = *E*(*t*) · *d* · cos*θ* is applied, where *d* is the size and *E*(*t*) is the stimulated electric field in vacuum that is equivalent to the macroscopic electric field multiplied by the frequency-dependent dielectric constant. Meanwhile, the transmembrane life ion flows and power dissipations are calculated accordingly by Eqs. (11) to (16).

Then, the relationships of the membrane potentials *V_m_*, transmembrane Na^+^ ion flows *J_Na_hp_* and *J_Na_ATPase_* and power dissipations *P_ATPase_* with the envelope *E_env_* of the terahertz unipolar picosecond pulse train are studied.

## Data Availability

All data generated or analyzed during this study are available from the corresponding authors on reasonable request.

## Supplementary Materials

Supplementary 1 Supplementary Notes Figs. S1 to S9
