# Supplementary material for: Study on the Effect of the Envelope of Terahertz Unipolar Stimulation on Cell Membrane Communication-Related Variables
Source: Research (Wash D C). 2025 Jul 15;8:0755. doi: 10.34133/research.0755 (PMC12260226; doi:10.34133/research.0755)
Supplement: Supplementary 1 — Supplementary Notes Figs. S1 to S9 [file research.0755.f1.docx]

Supplementary materials on “Study on effect of envelope of THz unipolar stimulation on cell membrane communication-related variables”

Wenfei Bo ^1^, Rong Che ^1^, Feng Jia ^1^, Kai Sun ^1^, Qiang Liu ^1^, Lemeng Guo ^1^, Xiaobo Zhang ^1^, and Yubin Gong ^2^

^1^ College of Information and Communication, National University of Defense Technology, Wuhan, 430000, China

^2^ School of Electronic Science and Engineering, University of Electronic Science and Technology of China, Chengdu, 611731, China

**1. Variations of ion concentrations for Na+ and K+ ions under THz unipolar stimulation with signals contained in envelope**

(a) (b)

(c) (d)

***Fig. S1.*** ***(a)*** *Variation of average intracellular ion concentrations of Na+ c_Na-i_* ***(a)*** *and K+ c_K-i_* ***(c)*** *and average extracellular ion concentrations of Na+ c_Na-o_* ***(b)*** *and K+ c_K-o_* ***(d)*** *in the case of Gauss envelopes with amplitudes of 0.9×10^7^, 2×10^7^ and 3.68×10^7^ V/m, and in the case of the envelopes of trapezoid and rectangular with amplitude of 2×10^7^ V/m.*

**2.** **The influence of conductance *g_1_* and diffusion coefficients *D_j_* on the effect of envelope of terahertz unipolar stimulation on cell membrane communication-related variables**

*2.1. The influence of conductance g_1_*

In order to investigate the effect of the conductance in eq. (8) on this study in case the conductance is changed and different under the terahertz stimulation, the variation rates of membrane potentials and the transmembrane life ion flows as well as the accompanying power dissipations are additionally simulated in the case that the conductance in eq. (8) is increased by a factor of 4. The terahertz unipolar stimulation is of Gaussian envelope at the amplitude of 2×10^7^ V/m. Figs. S2-S5 illustrate the membrane potentials, ion flows as well as their variation rates and accompanying power dissipations in the case that the conductance in eq. (8) is increased by a factor of 4.

By comparing Fig. S2(a)(b) and Fig. 2(b)(e), it can be seen that the membrane potentials as well as the membrane conductivity due to the hydrophilic pores are similar as the conductance *g*_1_ in eq. (8) is increased by a factor of 4. And the ion flow via hydrophilic pores is similar and the ion flow via Na+, K+-ATPase has about four times amplitudes (see Fig. S3(a)(b) and Fig. 5(b)7(b)). Most importantly, it is clear from Fig. S4(a)(b) and Fig. 3(b)5(d)(e) that the conductance in eq. (8) has insignificant influence on the shape of the envelope of the variation rates of membrane potentials and ion flows via hydrophilic pores, which is Gaussian and reflects the envelope of the terahertz unipolar stimulation. Thus, the relationships of the variation rates of membrane potentials and ion flow via hydrophilic pores with the envelope of the stimulation are the same when the conductance changes. Additionally, the variation rates of the ion flow via Na+, K+-ATPase seem irrelevant to the envelope of the terahertz unipolar stimulation and the power dissipations accompanied are around the level of 10^-11^ W (see Fig. S5(a)(b) and Fig. 8, 10). Thus, the relationships of the variation rates of ion flow via Na+, K+-ATPase and accompanying power dissipations with the envelope of the stimulation are the same when the conductance changes. As a consequence, the conductance in eq. (8) plays trivial roles in the effect of envelope of terahertz unipolar stimulation on cell membrane communication-related variables, and the changes in the conductance due to the effects of terahertz waves have insignificant influences.

(a) (b)

***Fig. S2.*** ***(a)****Membrane potentials at 𝜃 = 0, 𝜋/6, 𝜋/3, 𝜋/2, 2𝜋/3, 5𝜋/6 and 𝜋 versus time in the case of 6 ns, 0.8 THz unipolar stimulation with Gauss envelopes at the amplitude of 2×10^7^ V/m in the case that the conductance in eq. (8) is increased by a factor of 4.* ***(b)*** *Membrane conductivities at 𝜃 = 0, 𝜋/6, 𝜋/3, 𝜋/2, 2𝜋/3, 5𝜋/6 and 𝜋 versus time in the case of 6 ns, 0.8 THz unipolar stimulation with Gauss envelopes at the amplitude of 2×10^7^ V/m in the case that the conductance in eq. (8) is increased by a factor of 4.*

(a) (b)

***Fig. S3.*** ***(a)*** *The transmembrane Na+ flow via hydrophilic pores at 𝜃 = 0, 𝜋/6, 𝜋/3, 𝜋/2, 2𝜋/3, 5𝜋/6 and 𝜋 versus time in the case of 6 ns, 0.8 THz unipolar stimulation with Gauss envelopes at the amplitude of 2×10^7^ V/m in the case that the conductance in eq. (8) is increased by a factor of 4.* ***(b)*** *The transmembrane Na+ flow via Na+, K+-ATPase at 𝜃 = 0, 𝜋/6, 𝜋/3, 𝜋/2, 2𝜋/3, 5𝜋/6 and 𝜋 versus time in the case of 6 ns, 0.8 THz unipolar stimulation with Gauss envelope at the amplitude of 2×10^7^ V/m in the case that the conductance in eq. (8) is increased by a factor of 4.*

(a)

(b)

***Fig. S4.*** ***(a)*** *Variation rates of membrane potentials (MPs) at 𝜃 = 0, 𝜋/6, 𝜋/3, 𝜋/2, 2𝜋/3, 5𝜋/6 and 𝜋 versus time in the case of 6 ns, 0.8 THz unipolar stimulation with Gauss envelopes at the amplitude of 2×10^7^ V/m in the case that the conductance in eq. (8) is increased by a factor of 4.* ***(b)*** *Variation rates of the transmembrane Na+ flow via hydrophilic pores at 𝜃 = 0, 𝜋/6, 𝜋/3, 𝜋/2, 2𝜋/3, 5𝜋/6 and 𝜋 versus time in the case of 6 ns, 0.8 THz unipolar stimulation with Gauss envelopes at the amplitude of 2×10^7^ V/m in the case that the conductance in eq. (8) is increased by a factor of 4. Inset is the enlarge in vertical axes.*

(a)

(b)

***Fig. S5.*** ***(a)*** *Variation rates of the transmembrane Na+ flow via Na+, K+-ATPase at 𝜃 = 0, 𝜋/6, 𝜋/3, 𝜋/2, 2𝜋/3, 5𝜋/6 and 𝜋 versus time in the case of 6 ns, 0.8 THz unipolar stimulation with Gauss envelope at the amplitude of 2×10^7^ V/m in the case that the conductance in eq. (8) is increased by a factor of 4.* ***(b)*** *Average power dissipation of Na+, K+-ATPase in the case of Gauss envelopes with amplitudes of 2×10^7^ V/m in the case that the conductance in eq. (8) is increased by a factor of 4.*

*2.2. The influence of* *diffusion coefficients D_j_*

For the sake of investigating the effect of the diffusion coefficients *D*_j_ in eqs. (11) and (12) on this study in case the diffusion coefficients are changed and different under the terahertz stimulation, the variation rates of membrane potentials and the transmembrane life ion flows as well as the accompanying power dissipations are simulated in the case that the diffusion coefficients in eqs. (11) and (12) are increased by a factor of 4. The terahertz unipolar stimulation is of Gaussian envelope at 2×10^7^ V/m. And the membrane potentials, ion flows as well as their variation rates and accompanying power dissipations are illustrated in Figs. S6-S9.

By comparing Fig. S6(a)(b) and Fig. 2(b)(e), the membrane potential variations show the same before the activation of hydrophilic pores (around < 4.8 ns). After the activation, the membrane conductivities due to the pores increase and finally reach the relatively stable value which is a bit larger in the case that the diffusion coefficients in eqs. (11) and (12) are increased by a factor of 4. The reason why the final relatively stable value the membrane conductivity reaches is not four times larger is the non-linear relationships between the membrane conductivity *I_p_*/*V_m_* and the diffusion coefficients based on eqs. (8) and (11). And after the activation (around > 4.8 ns) the membrane potential decreases towards zero (*θ* = *π* in Fig. S6(a)) a bit faster. From the comparison of Fig. S7(a)(b) and Fig. 5(b)7(b), the amplitude of the ion flow via hydrophilic pores is a bit larger when the diffusion coefficients increase. Nevertheless, it is apparent from Fig. S8(a)(b) and Fig. 3(b)5(d)(e) that the diffusion coefficients in eqs. (11) and (12) have insignificant influence on the shape of the envelope of the variation rates of membrane potentials and ion flows via hydrophilic pores, which is Gaussian and reflects the envelope of the terahertz unipolar stimulation. Hence, the relationships of the variation rates of membrane potentials and ion flow via hydrophilic pores with the envelope of the stimulation are the same when the diffusion coefficients change. Additionally, the variation rates of the ion flow via Na+, K+-ATPase seem irrelevant to the envelope of the terahertz unipolar stimulation and the power dissipations accompanied are around the level of 10^-11^ W (see Fig. S9(a)(b) and Fig. 8, 10). Thus, the relationships of the variation rates of ion flow via Na+, K+-ATPase and accompanying power dissipations with the envelope of the stimulation are the same when the diffusion coefficients change. As a result, the diffusion coefficients in eqs. (11) and (12) play trivial roles in the effect of envelope of terahertz unipolar stimulation on cell membrane communication-related variables, and the changes in the diffusion coefficients due to the effects of terahertz waves have insignificant influences.


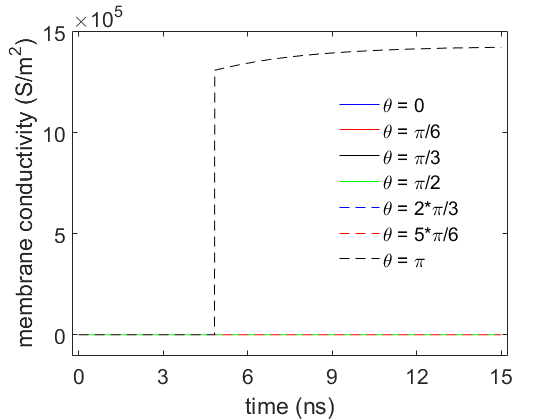


(a) (b)

***Fig. S6.*** ***(a)*** *Membrane potentials at 𝜃 = 0, 𝜋/6, 𝜋/3, 𝜋/2, 2𝜋/3, 5𝜋/6 and 𝜋 versus time in the case of 6 ns, 0.8 THz unipolar stimulation with Gauss envelopes at the amplitude of 2×10^7^ V/m in the case that the diffusion coefficients in eqs. (11) and (12) are increased by a factor of 4.* ***(b)*** *Membrane conductivities at 𝜃 = 0, 𝜋/6, 𝜋/3, 𝜋/2, 2𝜋/3, 5𝜋/6 and 𝜋 versus time in the case of 6 ns, 0.8 THz unipolar stimulation with Gauss envelopes at the amplitude of 2×10^7^ V/m in the case that the diffusion coefficients in eqs. (11) and (12) are increased by a factor of 4.*


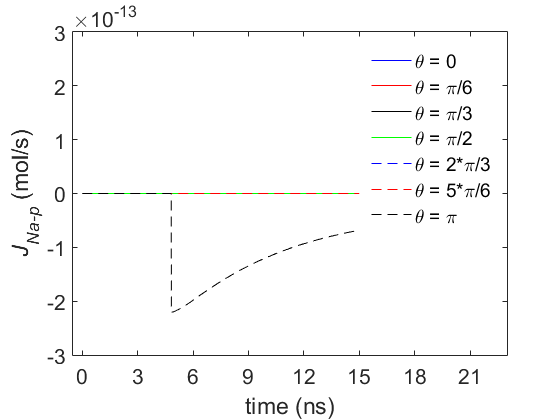

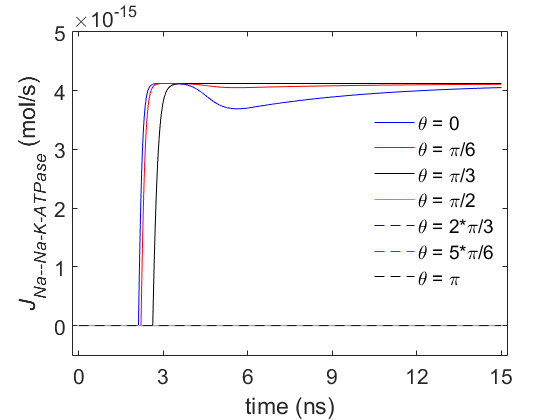


(a) (b)

***Fig. S7.*** ***(a)*** *The transmembrane Na+ flow via hydrophilic pores at 𝜃 = 0, 𝜋/6, 𝜋/3, 𝜋/2, 2𝜋/3, 5𝜋/6 and 𝜋 versus time in the case of 6 ns, 0.8 THz unipolar stimulation with Gauss envelopes at the amplitude of 2×10^7^ V/m in the case that the diffusion coefficients in eqs. (11) and (12) are increased by a factor of 4.* ***(b)*** *The transmembrane Na+ flow via Na+, K+-ATPase at 𝜃 = 0, 𝜋/6, 𝜋/3, 𝜋/2, 2𝜋/3, 5𝜋/6 and 𝜋 versus time in the case of 6 ns, 0.8 THz unipolar stimulation with Gauss envelope at the amplitude of 2×10^7^ V/m in the case that the diffusion coefficients in eqs. (11) and (12) are increased by a factor of 4.*


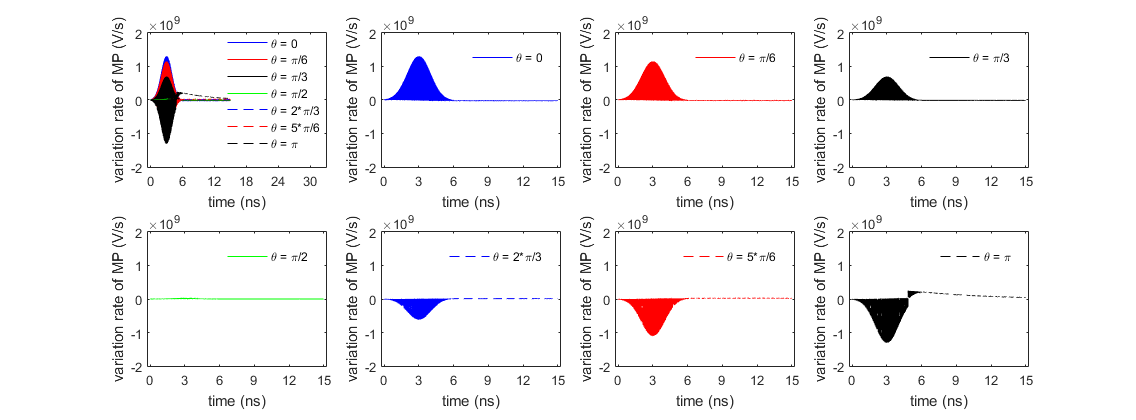


(a)

(b)

***Fig. S8.*** ***(a)*** *Variation rates of membrane potentials (MPs) at 𝜃 = 0, 𝜋/6, 𝜋/3, 𝜋/2, 2𝜋/3, 5𝜋/6 and 𝜋 versus time in the case of 6 ns, 0.8 THz unipolar stimulation with Gauss envelopes at the amplitude of 2×10^7^ V/m in the case that the diffusion coefficients in eqs. (11) and (12) are increased by a factor of 4.* ***(b)*** *Variation rates of the transmembrane Na+ flow via hydrophilic pores at 𝜃 = 0, 𝜋/6, 𝜋/3, 𝜋/2, 2𝜋/3, 5𝜋/6 and 𝜋 versus time in the case of 6 ns, 0.8 THz unipolar stimulation with Gauss envelopes at the amplitude of 2×10^7^ V/m in the case that the diffusion coefficients in eqs. (11) and (12) are increased by a factor of 4. Inset is the enlarge in vertical axes.*


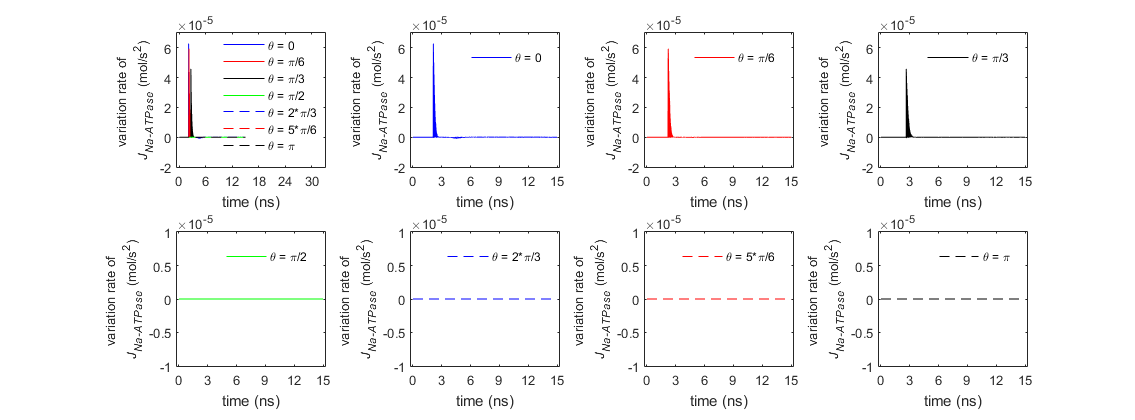


(a)


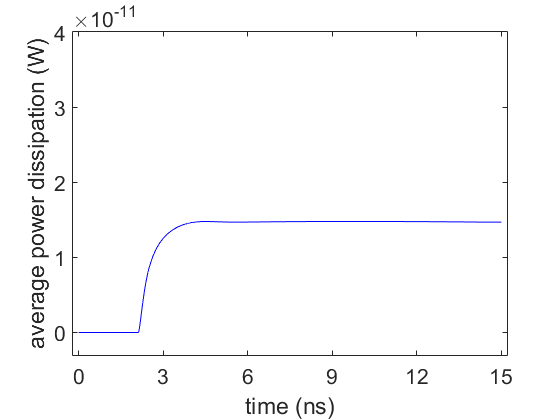


(b)

***Fig. S9.*** ***(a)*** *Variation rates of the transmembrane Na+ flow via Na+, K+-ATPase at 𝜃 = 0, 𝜋/6, 𝜋/3, 𝜋/2, 2𝜋/3, 5𝜋/6 and 𝜋 versus time in the case of 6 ns, 0.8 THz unipolar stimulation with Gauss envelope at the amplitude of 2×10^7^ V/m in the case that the diffusion coefficients in eqs. (11) and (12) are increased by a factor of 4.* ***(b)*** *Average power dissipation of Na+, K+-ATPase in the case of Gauss envelopes with amplitudes of 2×10^7^ V/m in the case that the diffusion coefficients in eqs. (11) and (12) are increased by a factor of 4.*
